# Cardiorespiratory Fitness and Long-Term Survival in “Low-Risk” Adults

**DOI:** 10.1161/JAHA.112.001354

**Published:** 2012-08-24

**Authors:** Carolyn E. Barlow, Laura F. DeFina, Nina B. Radford, Jarett D. Berry, Kenneth H. Cooper, William L. Haskell, Lee W. Jones, Susan G. Lakoski

**Affiliations:** The Cooper Institute, University of Texas Southwestern Medical Center, Dallas, TX (C.E.B., L.F.D.); The Cooper Clinic, University of Texas Southwestern Medical Center, Dallas, TX (N.B.R., K.H.C.); Department of Internal Medicine/Cardiology, University of Texas Southwestern Medical Center, Dallas, TX (J.D.B.); Stanford Prevention Research Center, Stanford University, Palo Alto, CA (W.L.H.); Duke Cancer Institute, Durham, NC (L.W.J.); Department of Internal Medicine, University of Vermont, Burlington, VT (S.G.L.)

**Keywords:** cardiorespiratory fitness, risk, low, Framingham Risk Score, cardiovascular disease

## Abstract

**Background:**

We sought to establish whether cardiorespiratory fitness had important implications for long-term cardiovascular risk among individuals classified as low risk by the Framingham Risk Score (10-year coronary heart disease risk <10%). Prognostic factors of long-term cardiovascular risk are needed for low-risk subjects who make up the largest percentage of the US population.

**Methods and Results:**

The study population was composed of men and women, 30 to 50 years of age, who had a baseline medical exam at the Cooper Clinic, Dallas, TX, between 1970 and 1983. Eligible individuals were defined as at low risk for coronary heart disease by Framingham Risk Score at the time of study entry and had no history of diabetes (n=11 190). Cardiorespiratory fitness was determined by maximum graded exercise treadmill tests. Over an average 27±2-year period, 15% of low-fit (quintile 1) compared to 6% of high-fit (quintile 5) individuals died (*P*<0.001). A 1–metabolic equivalent level increase in baseline fitness was associated with an 11% reduction in all-cause deaths and an 18% reduction in deaths due to cardiovascular disease (CVD) after adjustment for age, sex, body mass index, systolic blood pressure, total cholesterol, blood glucose levels, smoking, and early family history of coronary disease. There was an incremental decrease in CVD risk with increasing fitness quintile, such that the high fit had the lowest adjusted 30-year CVD mortality rate (hazard ratio 0.29, 95% CI: 0.16–0.51) compared to the low fit.

**Conclusions:**

Cardiorespiratory fitness is associated with a significant reduction in long-term CVD among individuals identified as low risk by Framingham Risk Score. These data suggest that preventive lifestyle interventions geared to optimize cardiorespiratory fitness, even among a “low-risk” subset, should be considered to improve CVD-free survival. **(J Am Heart Assoc. 2012;1:e001354 doi: 10.1161/JAHA.112.001354.)**

## Introduction

Framingham Risk Score (FRS) is a global risk score that estimates an individual's 10-year risk for a future coronary heart disease (CHD) event.^1^ With the FRS, individuals are categorized into 3 categories: *low risk*, corresponding to a 10% estimated risk for a CHD event over the next 10 years; *intermediate risk*, a 10% to 20% estimated risk for a CHD event over the next 10 years; or *high risk*, >20% estimated risk for a CHD event over the next 10 years. FRS is based on traditional risk factors, such as age, smoking history, cholesterol levels, and blood pressure. FRS is widely accepted as the “gold standard” for 10-year CHD risk assessment and is used in preventive health guidelines to inform clinicians about cut points at which to initiate medical therapy for untreated risk factors (eg, low-density lipoprotein cholesterol).^2^ Appropriately, FRS places significant emphasis on age as a risk factor for CHD, with treatment thresholds favoring older adults; consequently, younger patients with lower short-term risk are not given treatment priority despite high lifetime CHD risk.^3^

In light of this limitation, several research groups, including our own, have developed alternative classification systems, such as the Reynolds Risk Score, which adds C-reactive protein and family history to the traditional risk factors^4^ and includes use of computed tomography to detect coronary artery calcium^5,6^ as well as measurement of cardiorespiratory fitness with maximal exercise tests.^7^ Still, the vast majority of prior work in this area has focused on upstaging patients to appropriately reclassify individuals traditionally classified as low risk into an intermediate-risk category, with the clinical goal of early aggressive preventive therapy (eg, statins).^8^ Less emphasis has been given to patients who remain classified as low risk. This is an important subset, given that (1) these patients represent a large proportion of the population,^9^ and (2) low-risk patients do not necessarily stay low risk throughout their lifetimes.

Cardiorespiratory fitness is a robust predictor of cardiovascular disease (CVD)^10–12^ and incrementally improves the prediction of both short-term (10-year) and long-term (25-year) CVD mortality rate beyond traditional risk factors.^7^ However, whether cardiorespiratory fitness is prognostic of long-term CVD risk among individuals appropriately classified as low risk by FRS has not been investigated. Accordingly, here we evaluated the prognostic importance of cardiorespiratory fitness in 11 190 men and women classified as low risk by FRS in the Cooper Center Longitudinal Study.

## Methods

### Study Population and Medical Examination

Patients enrolled in The Cooper Center Longitudinal Study signed an informed consent form and approved of the use of their data for research. The data collection and informed consent are reviewed and approved annually by The Cooper Institute's Institutional Review Board.

The study population was composed of men and women, 30 to 50 years of age, who had a baseline medical exam at the Cooper Clinic between 1970 and 1983, were classified as low risk by FRS (10-year risk of CHD: <10%) at time of study entry, and had no history of diabetes mellitus. At the baseline Cooper Clinic visit, an extensive medical history was taken, and anthropometric measurements, laboratory analysis, and assessment of cardiorespiratory fitness by maximal exercise treadmill tests were performed. Information about age, sex, and health habits was obtained by questionnaires and physician verified. Body mass index (BMI) was calculated from measured weight and height. Blood pressure was measured with standard auscultatory methods after the participant had been seated for 5 minutes. Physical activity was assessed by a physical activity index variable composed of 4 categories: 0 = no regular physical activity; 1 = physical activities other than walking, jogging, or running; 2 = 0 to 10 miles/week walking, jogging, or running; 3 = 11 to 20 miles/week walking, jogging, or running; and 4 = >20 miles/week walking, jogging, or running. Systolic and diastolic blood pressure was recorded as the first and fifth Korotkoff sounds, respectively. A 12-hour fasting antecubital venous blood sample was obtained, and plasma concentrations of glucose and lipids were determined with automated bioassays in the Cooper Clinic laboratory, which meets quality control standards of the Centers for Disease Control and Prevention Lipid Standardization Program.

As reported previously, cardiorespiratory fitness was measured by a maximal treadmill exercise test that used a modified Balke protocol. In this protocol, treadmill speed is set initially at 3.3 mph (88 m/min). In the first minute, the grade is set at 0%, followed by 2% in the second minute and an increase of 1% for every minute thereafter. After 25 minutes, the grade remains unchanged, but the speed is increased 0.3 mph (5.4 m/min) for each additional minute until the test is terminated for volitional exhaustion. Time achieved on this protocol correlates directly with measured maximal oxygen uptake (*r*=0.92).^13^ With the use of well-characterized regression equations, treadmill times from the Balke protocol allow for estimation of fitness level in metabolic equivalents (METs).^14^ Estimates of cardiorespiratory fitness were expressed as both continuous and categorical variables. Individuals were classified into cardiorespiratory fitness quintiles based on age- and sex-specific strata.

### Ascertainment of Death

All participants in the total cohort were followed up from the date of their baseline examination until their date of death or until December 31, 2008. The National Death Index was the primary data source for death surveillance. Cardiovascular death was defined as *International Classification of Diseases, 9th revision* codes 390–459 for deaths before 1999 or *10th revision* codes I01–I99 for deaths occurring between 1999 and 2008.

### Statistical Methods

Descriptive means and standard deviations were calculated for the continuous independent and dependent variables, with frequencies and proportions for categorical measures. Differences in means and proportions of outcome measures between groups stratified by risk factor status were assessed by χ^2^ test or analysis of variance, respectively. Baseline characteristics were compared across quintiles of cardiorespiratory fitness by using the Jonckheere-Terpstra nonparametric trend test. Cox proportional-hazards regression was used to estimate hazard ratio (HRs) and 95% confidence intervals (CIs) adjusted for age, sex, BMI, glucose, smoking status, systolic blood pressure, total cholesterol, and family history of premature CHD. We also examined multiplicative interactions for cardiorespiratory fitness and sex, as well as cardiorespiratory fitness and BMI, by including their cross-products in the statistical models. Analyses were performed in SAS version 9.2 (SAS Institute Inc, Cary, NC). All significance testing was 2 sided, with a *P* value <0.05 considered statistically significant.

## Results

The average age of the cohort at baseline was 41±4 years, with a mean BMI of 24.8±3.5 kg/m^2^ and FRS of 5±2% for men and 3±2% for women. The mean FRS and proportion of individuals with FRS <6% versus 6% to 9% varied by cardiorespiratory fitness quintile (*P*<0.001). Individuals with low cardiorespiratory fitness (Q1) had an average MET level of 8.7±0.9 for men and 6.6±0.7 for women; high-fit (Q5) individuals had a MET level of 14.8±1.5 for men and 11.5±1.1 for women (Table 1). Seventy-three percent of low-fit individuals did not participate regularly in physical activity, as compared to 15% of the high fit. Increasing dose of physical activity was associated with higher levels of cardiorespiratory fitness (*P*<0.001). Cardiorespiratory fitness quintile was associated inversely with BMI, total cholesterol, blood pressure, fasting glucose, and smoking (*P*<0.001 for all), though a majority of the cardiovascular risk factors were within the normal range in each fitness quintile. A family history of premature CHD was present in 1.4% of low-fit and 0.4% of high-fit patients (*P*<0.001).

**Table 1.** Baseline Characteristics of Individuals Classified as Low Risk by FRS According to Cardiorespiratory Fitness Quintile (n=11 190)

	Fitness Quintiles					
	Q1 (n=2072)	Q2 (n=2191)	Q3 (n=2369)	Q4 (n=2294)	Q5 (n=2264)	*P*
Age, y, mean±SD	40.6±4.2	41.1±4.4	40.5±4.3	40.7±4.3	40.6±4.4	0.02

Follow-up time, y, mean±SD	27.5±5.2	27.2±4.6	27.4±4.1	26.9±4.3	26.5±3.9	<0.001

FRS:						<0.001

mean±SD	5±2	5±2	5±2	4±2	4±2	

n (%)						

<6%	1197 (58)	1365 (62)	1603 (68)	1662 (72)	1854 (82)	

6% to 9%	875 (42)	826 (38)	766 (32)	632 (28)	410 (18)	

Cardiorespiratory fitness, METs, mean (95% CI)						<0.001

Men	8.7 (4.4–9.9)	10.2 (9.4–10.8)	11.4 (10.3–12.2)	12.6 (11.3–13.5)	14.8 (12.6–22.5)	

Women	6.6 (4.4–7.6)	7.7 (6.3–8.5)	8.7 (8.1–9.4)	9.5 (8.5–10.3)	11.5 (9.9–18.3)	

BMI, kg/m^2^, mean±SD						<0.001

Men	27.9±4.3	26.3±2.9	25.4±2.5	24.9±2.3	23.9±2.0	

Women	24.4±5.1	22.5±3.2	21.9±2.8	21.3±2.2	20.6±1.6	

Physical activity, n (%)						<0.001

No organized physical activity	1520 (73)	1264 (58)	1057 (45)	611 (26)	349 (15)	

Nonrunning activities	309 (15)	444 (20)	466 (20)	373 (16)	231 (10)	

Running 0 to 10 miles/week	221 (11)	430 (20)	737 (31)	979 (43)	749 (33)	

Running 11 to 20 miles/week	16 (0.7)	49 (2)	92 (4)	265 (12)	497 (22)	

Running >20 miles/week	6 (0.3)	4 (0.2)	17 (0.7)	67 (3)	438 (20)	

Total cholesterol, mg/dL, mean±SD	205.5±33.8	205.1±34.4	202.9±33.3	200.0±32.9	195.5±30.0	<0.001

Systolic blood pressure, mm Hg, mean±SD	119.0±13.0	116.5±12.5	116.2±12.2	115.2±12.5	115.4±12.6	<0.001

Diastolic blood pressure, mm Hg, mean±SD	79.9±9.0	78.6±8.7	77.8±8.3)	76.9±8.6	76.5±8.2	<0.001

Fasting glucose, mg/dL, mean±SD	98.9±17.3	97.6±13.6	96.5±10.5	95.6±10.0	95.5±9.8	<0.001

Family history of premature CHD, n (%)	29 (1.4)	14 (0.6)	10 (0.4)	7 (0.3)	10 (0.4)	<0.001

Smoker, n (%)	499 (24)	424 (19)	363 (15)	272 (12)	165 (7)	<0.001

Values are given as mean ± standard deviation or number (percentage) of patients, as noted.

*Running, jogging, or walking activities.

**Figure 1.**
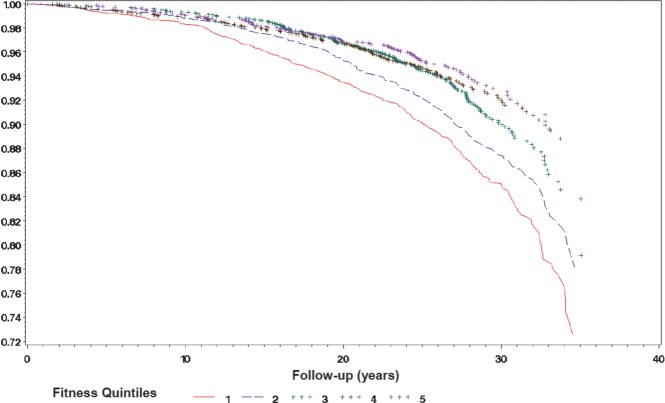
Thirty-year survival by cardiorespiratory fitness quintile among individuals classified as low risk by FRS at 30 to 50 years of age. Cardiorespiratory fitness quintiles (Q) were based on age- and sex-specific strata. Red line indicates Q1 (n=2072); blue line, Q2 (n=2191); green line, Q3 (n=2369); black line, Q4 (n=2294); and purple line, Q5 (n=2264).

The probability of survival by cardiorespiratory fitness quintile is illustrated in Figure 1. Over an average 27±2-year period, 774 individuals died: 15% low-fit (Q1) individuals compared to 6% high-fit (Q5) individuals (*P*<0.001; Table 2). Absolute mortality rates were 16% versus 6% for low- and high-fit men (*P*<0.001), respectively, and 10% versus 4% for low- and high-fit women (*P*=0.006), respectively. When limited to nonsmokers or individuals with baseline FRS ≤6%, associations between fitness and death were similar to the overall cohort. For example, among individuals with a baseline FRS <6%, 12% of low-fit versus 5% of high-fit individuals died at 30 years (*P*<0.001). When absolute CVD mortality rate was assessed, 5.2% of low-fit versus 0.8% of high-fit individuals died from a cardiovascular cause (*P*<0.001).

**Table 2.** Thirty-Year All-Cause Deaths and CVD Deaths by Cardiorespiratory Fitness Quintile Among Individuals Classified as Low Risk by FRS at 30 to 50 Years of Age

	Fitness Quintiles					
	Q1	Q2	Q3	Q4	Q5	*P*
Crude deaths, n (%)						

Overall	306 (15)	249 (11)	199 (8)	156 (7)	130 (6)	<0.001

Men	260 (16)	205 (13)	166 (9)	122 (7)	108 (6)	<0.001

Women	46 (10)	44 (8)	33 (7)	34 (6)	22 (4)	=0.006

Nonsmokers	211 (13)	202 (11)	164 (8)	134 (7)	123 (6)	<0.001

FRS <6% over 10 years	138 (12)	126 (9)	113 (7)	86 (5)	98 (5)	<0.001

Crude CVD deaths, n (%)	107 (5.2)	70 (3.2)	61 (2.5)	38 (1.7)	19 (0.8)	<0.001

**Figure 2.**
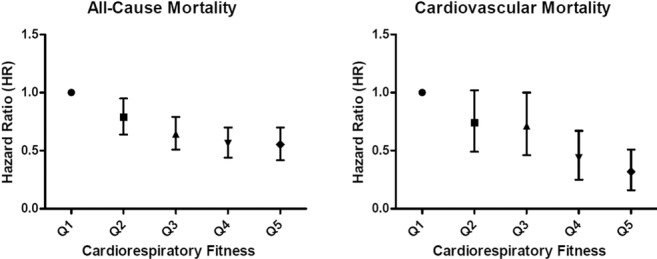
Adusted 30-year mortality risk and cardiovascular death by fitness quintile (Q) among individuals classified as low risk by FRS at 30 to 50 years of age. Adjusted for age, sex, BMI, systolic blood pressure, total cholesterol, blood glucose, smoking, and family history of early CHD (<50 years of age).

Table 3 shows the adjusted 30-year mortality and CVD risks associated with a 1-MET change in cardiorespiratory fitness. A 1–MET level increase in baseline fitness was associated with an 11% reduction in all-cause deaths and an 18% reduction in CVD deaths after adjustment for age, sex, BMI, systolic blood pressure, total cholesterol, blood glucose levels, smoking, and family history of early coronary disease. Figure 2 demonstrates adjusted HRs by fitness quintile for all-cause death and CVD death. Compared to Q1, individuals in Q2 (HR 0.78, 95% CI: 0.64–0.95), Q3 (HR 0.63, 95% CI: 0.51–0.79), Q4 (HR 0.40, 95% CI: 0.44–0.70), and Q5 (HR 0.54, 95% CI: 0.42–0.70) had lower mortality risk. In addition, there was an incremental decrease in CVD risk with increasing fitness quintile, such that high-fit patients had the lowest 30-year CVD mortality rate (HR 0.29, 95% CI: 0.16–0.51) compared to the low fit. Neither sex nor BMI modified the relationship between cardiorespiratory fitness and CVD outcomes (*P*>0.1 for both).

**Table 3.** Adjusted* 30-Year All-Cause Death Risk and CVD Death Risk by 1-MET Increase in Cardiorespiratory Fitness Among Individuals Classified as Low Risk by FRS at 30 to 50 Years of Age

	All-Cause Mortality (n=774)	Cardiovascular Mortality (n=214)
Cardiorespiratory fitness	0.89 (0.86–0.93), *P*<0.001	0.82 (0.76–0.89), *P*<0.001

(1-MET increase)	11% reduction	18% reduction

Values given as HR (95% CI).

* Adjusted for age, sex, BMI, systolic blood pressure, total cholesterol, blood glucose, smoking, and family history of early CHD (<50 years of age).

## Discussion

Among individuals classified as low risk by FRS in midlife (40 years of age), a 1-MET increase in cardiorespiratory fitness resulted in an 18% reduction in cardiovascular mortality over 30 years. Avoiding low fitness (MET level <9 in men and <7 in women) was associated with a significantly lower risk of all-cause and CVD death. Compared to the low fit, individuals in the highest quintile of cardiorespiratory fitness had the greatest protection from 30-year CVD death (HR 0.29, 95% CI: 0.16–0.51). These data suggest that preventive lifestyle interventions geared to optimize cardiorespiratory fitness, even when individuals are low risk in midlife, should be considered to improve CVD-free survival.

More than 100 million adults, including 95% of women and 66% of men in the United States, are low risk according to FRS.^9^ Despite use of reclassification tools, a large majority of individuals appropriately remain or are reclassified as low risk. For example, measurement of coronary artery calcium in low-risk women results in only a small proportion of women (5% with coronary artery calcium ≥300 Agatston units) being reclassified into a risk category >10% extrapolated to 10 years.^6^ The study did not take into account the additional number of patients downgraded to a low-risk status, a focus of a follow-up study.^15^ Use of the Reynolds Risk Score, which incorporates standard risk factors plus C-reactive protein and history of premature CHD, results in a net reclassification of 30% among low-risk individuals, with a majority (20%) downgraded to very low risk (10-year risk: <5%) and only 10% to a higher intermediate-risk classification (10-year risk: 10% to 20%).^4^ In previously published work by our group, cardiorespiratory fitness improved reclassification (into higher or lower risk categories) compared to traditional risk factors alone.^7^ The above studies, however, did not address the remaining low-risk population who would not be eligible for more aggressive preventive therapies (eg, aspirin, statins) on the basis of low-risk status. Thus, elucidating factors that add prognostic value to long-term survival and CVD in a low-risk FRS population is vitally important.

In the present study, we demonstrated that a 1-MET increase in low-risk patients resulted in an 18% reduction in long-term CVD mortality after traditional risk factors were accounted for, with high-fit individuals achieving the greatest protection. This protective effect was similar to data from Myers et al^16^ that showed a 12% reduction in mortality for every 1-MET increase in fitness among individuals referred for exercise testing (a higher-risk subset) over a 6-year period.^16^ Our data are of additional importance, given that a 1-MET increase was associated with reductions in 30-year mortality rate, a potentially more informative end point for low-risk individuals in midlife.

With regard to limitations of the present study, we studied a healthy white population at baseline, and thus generalizability to other subgroups cannot be made. It is important to note, however, that a main tenet of the study was that even healthy, low-risk individuals are negatively affected by low levels of cardiorespiratory fitness. The study is observational in nature, and thus causality cannot be inferred. It is possible that other unmeasured confounders among fit individuals (eg, access to preventive services, greater awareness of healthy habits, different levels of emotional and physical stress as well as self-motivation) affect associations between cardiorespiratory fitness and survival. In addition, an examination of relationships between cardiorespiratory fitness and other markers of CVD (eg, C-reactive protein, waist circumference, nutritional intake) was not possible given a lack of measurement or routine assessment of these factors during the study period.

The present study has important implications for clinical practice with regard to gearing exercise prescription (dose/intensity) to improve cardiorespiratory fitness among low-risk individuals. Indeed, physical activity is an important focus for primary prevention, with regular participation in moderate to vigorous physical activity recommended across different risk groups.^17–20^ On the basis of the present study, regular physical activity^17^ and higher doses of aerobic exercise to obtain greater cardiorespiratory fitness levels should be considered in low-risk individuals.
